# Sleep Stage Classification During CPAP Therapy from CPAP-Airflow and Wearable Fingertip Signals

**DOI:** 10.3390/s26123720

**Published:** 2026-06-11

**Authors:** Hsin-Yu Chen, Aatif Husain, Andrey V. Zinchuk, Henry K. Yaggi, Muneeb Ahsan, Cheng-Yao Chen, Shirah Pokusa, Hau-Tieng Wu

**Affiliations:** 1 PranaQ Pte. Ltd., Singapore 018937, Singapore; rebeccachen@pranaq.com; 2 Department of Neurology, Duke University Medical Center, Durham, NC 27704, USA; aatif.husain@duke.edu (A.H.);; 3 Department of Medicine, Yale University School of Medicine, New Haven, CT 06510, USA; andrey.zinchuk@yale.edu (A.V.Z.); henry.yaggi@yale.edu (H.K.Y.); muneeb.ahsan@yale.edu (M.A.); 4 PranaQ, Co., New York, NY 10004, USA; jerry.chen@pranaq.com; 5 Courant Institute of Mathematical Sciences, New York University, New York, NY 10012, USA

**Keywords:** continuous positive airway pressure, photoplethysmography, probabilistic ensembling, deep learning, convolutional neural network, sleep stage classification, home sleep monitoring

## Abstract

Background: Continuous Positive Airway Pressure (CPAP) therapy is the standard treatment for obstructive sleep apnea–hypopnea syndrome (OSAHS), and photoplethysmography (PPG) sensors are commonly used in wearable devices for home sleep apnea testing. The recorded airflow and PPG signals from both sensors capture rich physiological patterns. We hypothesize that by combining information from these signals, we can efficiently estimate sleep dynamics of patients receiving CPAP treatment. Methods: The airflow signals were obtained from CPAP titration devices, denoted as CPAP-airflow, while the PPG signals were collected using the PranaQ TipTraQ (TTQ001), a fingertip-worn wearable device. We separately trained one-dimensional convolutional neural networks for CPAP-airflow and PPG signals and fused their outputs through probabilistic ensembling to predict sleep stages. The ensemble method is a late-fusion soft-voting scheme that computes a linearly weighted combination of synchronized softmax probability vectors from the modality-specific models. Results: For three-stage classification (Wake, REM, NREM), the PPG-based and CPAP-airflow-based models achieved overall Cohen’s kappa scores of 0.511 and 0.452, respectively, while the ensembled model improved the overall kappa to 0.587. The F1-score for the REM stage improved to 0.706 using the ensemble method, compared to 0.685 and 0.532 achieved by the individual models, respectively. In the four-stage classification (Wake, REM, Light, Deep) task, a deep sleep sensitivity of 0.596 was attained through the application of probabilistic ensembling. Conclusions: A fusion scheme of complementary information from the CPAP and PPG enhances the accuracy of sleep stage detection and hence enables more precise sleep monitoring, especially with an improved REM identification. Clinical implications include applying the proposed algorithm to improve in-home auto-CPAP titration by capturing REM-related respiratory instability and avoiding under-titration in REM-dominant OSAHS, better reflecting the patient’s true nocturnal respiratory needs.

## 1. Introduction

Sleep plays a fundamental role in maintaining physiological homeostasis and cognitive function [1], and accurate characterization of sleep architecture is critical for the diagnosis and management of sleep disorders. Polysomnography (PSG) remains the clinical gold standard for sleep staging [2], as it simultaneously records multiple physiological signals, including electroencephalography (EEG), electrooculography (EOG), and electromyography (EMG). Despite its diagnostic value, PSG is resource-intensive, requires specialized equipment and clinical supervision, and is impractical for long-term or large-scale monitoring in home settings. These limitations have motivated substantial interest in alternative sleep monitoring approaches that rely on fewer, more accessible sensors while enabling scalable and non-invasive assessment.

In recent years, wearable devices have become increasingly prevalent in homecare environments. Such devices commonly incorporate photoplethysmography (PPG) sensors, which enable extraction of hemodynamic, cardiovascular and respiratory biomarkers, including peripheral oxygen saturation, heart rate variability (HRV), and pulse amplitude modulation [3], and inertial measurement units (IMUs) that provide information on body movement and physical activity [4]. Both PPG [5,6] and IMU sensors [4] encode rich sleep information. In addition, wearable modalities may include, but are not exclusively electrocardiography (ECG), EEG, EMG, or respiratory chest bands. Although recent studies have demonstrated the feasibility of sleep stage estimation using single-modality signals, such as PPG, IMU, or respiratory measurements, it is well recognized that single-channel approaches, while unobtrusive and cost-effective, are inherently more susceptible to environmental artifacts commonly encountered in home settings [7] and are more sensitive to inter-individual physiological variability [8]. Consequently, when multiple sensing modalities are available, leveraging multimodal information generally yields more robust and reliable sleep staging performance. Comprehensive reviews summarizing recent advances in wearable and multimodal sleep staging can be found in [9].

In this study, rather than developing yet another generic sleep staging model, we focus on a clinically motivated problem: the development of a feasible sleep staging framework tailored specifically for patients undergoing continuous positive airway pressure (CPAP) therapy, which is the standard treatment for obstructive sleep apnea–hypopnea syndrome (OSAHS) [10]. CPAP therapy has been shown to improve cognitive performance [11] and alter sleep architecture and sleep dynamics [11,12], with well-documented changes in EEG spectral properties and sleep stability following treatment initiation [13,14]. Modern CPAP machines continuously record airflow, hereafter referred to as CPAP-airflow, as well as delivered airway pressure throughout the night. It has been well known that airflow signals encode rich sleep dynamics [15,16] by reflecting upper-airway mechanics and ventilatory control, thereby providing physiologically meaningful information related to sleep–wake dynamics. This observation has motivated recent efforts to explore the feasibility of automatic sleep staging using CPAP-derived airflow signals alone or in combination with other modalities [17]. Nevertheless, sleep staging under CPAP presents unique challenges. The externally imposed positive airway pressure may introduce signal artifacts and alter baseline physiological states, potentially confounding conventional sleep stage signatures [18]. As a result, sleep staging in CPAP-treated patients is intrinsically more challenging than in untreated or healthy populations, underscoring the need for dedicated modeling strategies.

A natural question that arises is whether sleep staging performance can be improved when a patient undergoing CPAP therapy concurrently wears a minimally obtrusive fingertip-wearable device equipped with PPG and IMU sensors, compared with relying on CPAP-airflow signals alone. To address this question, we propose a dual-model framework that jointly leverages CPAP-airflow, PPG, and IMU signals for automatic sleep stage classification. Two modality-specific models were trained independently using data acquired from different devices, and their predicted class probabilities were subsequently fused using an ensemble strategy to produce the final sleep stage estimates. The proposed ensemble approach was evaluated on data collected at Duke University Hospital using two pretrained models. Our results demonstrate that appropriately combining heterogeneous physiological signals yields improved sleep staging performance compared with using either CPAP-derived signals or wearable-based signals alone.

The primary contributions and novelties of this work are summarized as follows:

- Clinical Specialization: We propose a sleep staging framework specifically tailored for the unique physiological conditions of patients undergoing CPAP therapy, addressing the challenges posed by positive airway pressure artifacts.
- Multimodal Synergy: We demonstrate that the integration of CPAP-airflow with fingertip-worn PPG and IMU signals significantly outperforms single-modality approaches, particularly in identifying REM sleep, which is critical for titration accuracy.
- Robust Fusion Architecture: We implement a late-fusion soft-voting ensemble strategy that effectively harmonizes heterogeneous data sources with different sampling resolutions and sensor characteristics.
- Practical Feasibility: Our approach leverages existing clinical hardware (CPAP) and minimally obtrusive wearables, offering a scalable solution for high-fidelity, in-home sleep monitoring without the need for full PSG.

The remainder of this paper is organized as follows. The Related Work section provides a comprehensive review of the recent literature focusing on PPG-based, respiration-based, and multimodal sleep monitoring approaches, with a summary of the state of the art presented in Table 1 and Table 2. Section 2 describes the datasets utilized for both model training and performance evaluation. In Section 3, we detail the signal pre-processing and feature extraction pipelines for CPAP and PPG + IMU signals, followed by an exposition of the proposed dual-model fusion architecture and the subsequent post-processing stages. Section 4 presents the experimental results for two-, three-, and four-stage sleep classification, featuring a comparative performance analysis of individual versus ensembled modalities, including direct inference and cross-validation results, and a confidence analysis of the ensemble output probabilities. Section 5 discusses the strengths and clinical implications of our findings, acknowledges the study’s limitations, and suggests directions for future research. Finally, Section 6 offers concluding remarks.

### Related Work

Automatic sleep staging has been extensively investigated using various physiological signal modalities, particularly in home-based systems with reduced-channel configurations. Among these, PPG-based approaches have gained increasing attention due to their compatibility with wearable devices and their ability to capture key physiological features, such as heart rate variability, blood oxygen saturation, and pulse amplitude, that are directly associated with sleep dynamics. Several studies have demonstrated that PPG-derived features can effectively estimate sleep–wake transitions and distinguish between REM and NREM stages, and in some cases, even differentiate light from deep sleep [19,20,21,22,23]. These investigations have employed deep learning techniques, including recurrent neural networks and convolutional neural networks (CNNs), which take feature time-series channels as the input. For example, Strumpf et al. [24] used PPG data from the Belun Ring to perform three-stage sleep classification with a CNN model pretrained on the large, open-access Sleep Heart Health Study (SHHS) dataset.

**Table 1:** Summary of three-stage classification performance from related work. ACTG: Actigraphy, ANN: Artificial Neural Network, LD: Linear Discriminant, NN: Neural Network, RE: Respiratory Effort, RF: Respiratory Flow, RIP: Respiratory Inductance Plethysmography, RNN: Recurrent Neural Network, SVM: Support Vector Machine, Temp: Temperature, XGB: eXtreme Gradient Boosting. The symbol “✓” indicates that the device utilized in the respective study is FDA-approved.

Reference	FDA Approved	Signal	Method	Overall			Sensitivity		
				Acc	κ	F1	Wake	REM	NREM
Long et al., 2014 [25]		RE	LD	0.762	0.45	-	-	-	-
Tataraidze et al., 2015 [26]		RIP	Bagging	0.804	0.65	-	-	-	-
Yang et al., 2016 [27]		RF	Classifier	0.74	0.49	-	-	-	-
Chen et al., 2023 [28]		RIP	XGB	0.76	0.54	0.677	0.749	0.762	0.769
Chung et al., 2024 [29]		RF	XGB	0.709	0.458	0.736	0.590	0.734	-
Chen et al., 2025 [17]		RF	CNN	0.785	0.605	0.727	0.748	0.604	0.838
Manjunath et al., 2025 [30]		RF	XGB	-	-	-	0.719	0.645	0.665
Wu et al., 2020 [20]		PPG	ANN + SVM	0.78	0.54	-	-	-	-
Korkalainen et al., 2020 [21]		PPG	CNN + RNN	0.801	0.65	-	0.72	0.70	0.87
Huttunen et al., 2021 [23]		PPG	CNN + RNN	0.833	0.72	0.83	0.75	0.86	0.89
Strumpf et al., 2023 [24]	✓	PPG	Transformer + CNN	0.80	0.606	0.741	0.58	0.70	0.90
Chen et al., 2025 [31]	✓	PPG + ACTG	NN	-	-	-	-	-	-
Sharan et al., 2025 [32]		PPG	NN	-	0.662	0.770	0.825	0.828	0.843
Zhang et al., 2024 [33]		ECG + PPG + ACTG + Temp	NN	0.84	0.729	0.842	0.862	0.823	0.820
Kazemi et al., 2024 [34]		RF + RIP + PPG	CNN+RNN	0.83	0.66	0.82	0.677	0.687	0.914
Krauss et al., 2025 [35]		ACTG + ECG + RIP	RNN	-	-	-	0.685	0.511	0.895

**Table 2:** Summary of four-stage classification performance from related work. ACTG: Actigraphy, ANN: Artificial Neural Network, KNN: K-Nearest Neighbors, LD: Linear Discriminant, NN: Neural Network, RE: Respiratory Effort, RF: Respiratory Flow, RIP: Respiratory Inductance Plethysmography, RNN: Recurrent Neural Network, SVM: Support Vector Machine, Temp: Temperature, XGB: eXtreme Gradient Boosting.

Reference	Signal	Method	Overall			Sensitivity			
			Acc	κ	F1	Wake	REM	Light	Deep
Long et al., 2014 [25]	RE	LD	0.638	0.38	-	-	-	-	-
Chang et al., 2018 [36]	IMU + Mic	KNN	-	-	-	-	0.700	0.696	0.598
Chen et al., 2023 [28]	RIP	XGB	0.65	0.458	0.544	-	-	-	-
Wu et al., 2020 [20]	PPG	ANN + SVM	0.62	0.41	-	0.526	0.659	0.625	0.596
Korkalainen et al., 2020 [21]	PPG	CNN + RNN	0.685	0.54	-	0.73	0.67	0.71	0.52
Huttunen et al., 2021 [23]	PPG	CNN + RNN	0.741	0.64	0.745	0.77	0.83	0.79	0.57
Sharan et al., 2025 [32]	PPG	NN	-	0.647	0.738	0.714	0.812	0.721	0.839
N. Sridhar et al., 2020 [22]	ECG	CNN	0.72	0.55	-	0.74	0.76	0.76	0.48
Zhang et al., 2024 [33]	ECG + PPG + ACTG + Temp	NN	0.753	0.615	0.741	0.840	0.798	0.615	0.733
Kazemi et al., 2024 [34]	RF + RIP + PPG	CNN + RNN	0.798	0.70	0.798	0.716	0.845	0.825	0.774
Han et al., 2024 [37]	In-ear Sound	Multi-class SVM	-	-	-	-	0.773	0.653	0.623

Recently, respiration-based sleep staging has attracted increasing attention due to the strong physiological relevance of respiratory dynamics to sleep regulation. Traditional time-domain and frequency-domain features characterizing breathing patterns have been widely used for sleep stage estimation [25,26,27]. More recently, Chung et al. [29] and Manjunath et al. [30] extracted topological features from airflow signals, though these features are fundamentally derived from time-domain representations, to infer sleep states. Chen et al. [28] adopted a similar strategy to characterize respiratory effort patterns from respiratory inductance plethysmography (RIP) belt signals for automated sleep staging. Krauss et al. [35] applied a deep learning approach, with respiratory rate variability from RIP, heart rate variability from ECG, and physical activity from IMU as inputs, to demonstrate the benefit of incorporating more information in the prediction model. In addition, CPAP-airflow has recently been explored for sleep stage estimation [17]. Collectively, these studies demonstrate the strong and informative relationship between respiratory dynamics and sleep stages.

While PPG-based and respiration-based approaches have shown promising performance, it is well recognized that relying on a single signal channel is more susceptible to noise and inter-individual variability. For example, the reliability of PPG signals can be compromised by motion artifacts or peripheral circulation fluctuations, limiting their robustness in real-world conditions. On the other hand, different physiological channels contain complementary information, and integrating multiple modalities has the potential to improve robustness and enhance discrimination of subtle sleep stage transitions. Accordingly, recent studies have increasingly explored multi-channel signal fusion for sleep stage classification using wearable devices. For example, Chen et al. [31] proposed a CNN model using finger PPG and IMU signals from a fingertip device for sleep and awake classification; Sharan et al. [32] proposed a lightweight neural network using PPG and IMU signals from a smart ring; Zhang et al. [33] proposed a deep learning approach for three-, four-, and five-stage sleep staging using chest ECG, triaxial accelerometry, and finger PPG; and Kazemi et al. [34,38] proposed a one-dimensional vision transformer for five-stage sleep classification using finger PPG signals along with respiratory flow and respiratory effort from an abdominal belt, among many others. We refer readers to review articles [7,9,39] for more information.

Beyond conventional wearables, recent research has explored the use of ubiquitous and non-contact sensors to minimize user burden. For instance, Han et al. [37] proposed EarSleep, which utilizes in-ear acoustic sensing to capture both physical and physiological activities for sleep stage detection. Similarly, Chang et al. [36] developed SleepGuard, a framework that leverages multi-sensor data from standard smartwatches, including IMU and microphones, to monitor sleep patterns, especially sleep posture and movements, and breathing events in a non-invasive manner. While these ubiquitous solutions provide potential accessibility for general health tracking, their performance and reliability in clinical populations, particularly those undergoing medical interventions like CPAP therapy, remain underexplored.

We are specifically interested in the situation when users are receiving CPAP treatment and have an extra mobile device with PPG and IMU installed. We summarized the three-stage and four-stage performance of the existing related work in Table 1 and Table 2, where we only report results using similar sensors as our framework. To our knowledge, how to establish an automatic sleep staging system, especially for REM and deep sleep detection, has not been well explored; as such, this gap is the focus of this paper.

## 2. Materials and Dataset Statistics

The dataset comprises 36 subjects who underwent split-night PSG with CPAP titration using a ResMed S9 VPAP Tx device (ResMed, San Diego, CA, USA) at Duke University Hospital (hereafter referred to as the Duke dataset). Participants wore the TipTraQ device (PranaQ Pte. Ltd., Singapore, Singapore; version: TTQ001) concurrently with standard PSG instrumentation throughout the entire study night. All participants were at least 20 years of age and had suspected OSAHS confirmed during the study. Exclusion criteria included a history of heart transplantation; heart failure with New York Heart Association (NYHA) class III or IV; chronic obstructive pulmonary disease with Global Initiative for Chronic Obstructive Lung Disease (GOLD) stage 3 or 4; tracheostomy; chronic opioid use; or severe stroke with a modified Rankin Scale (mRS) score ≥ 4. The study protocol was reviewed and approved by the Duke University Health System Institutional Review Board, and all participants provided written informed consent prior to enrollment. Recordings had a minimum duration of 2.5 h, with a mean duration of 4.2 h. Ground-truth sleep stages were annotated by certified PSG technologists, in accordance with the American Academy of Sleep Medicine (AASM) scoring manual [2].

Among the 36 recordings, four were excluded: one due to a total sleep time (TST) of less than 60 min, and three due to poor data quality with more than 50% of the total recording time corrupted, resulting in 32 recordings included in the final analysis. Across the remaining participants, the mean TST was 197.9 ± 54.6 min, with 46.5 ± 23.4 min in REM sleep, 151.1 ± 43.9 min in NREM sleep, and, specifically noting, 8.9 ± 15.9 min in deep sleep. Two subjects exhibited no REM sleep, while twenty-four subjects showed no deep sleep stages. The overall demographic and clinical characteristics of this dataset are shown in Table 3. The dataset includes subjects with moderate to severe sleep apnea, with an average Apnea–Hypopnea Index (AHI) exceeding 40 events per hour.

### 2.1. Benchmark

Given that much of the current state-of-the-art work focuses on either PPG or respiratory signals alone, we included both signal modalities to establish baseline comparisons. Accordingly, two models were trained separately using data from TipTraQ and CPAP, respectively, and their performances were used as benchmarks.

- TipTraQ Models: Trained using PPG and IMU signals to predict sleep stages, including two-stage (wake/sleep), three-stage (wake/REM/NREM), and four-stage (wake/REM/light/deep) classification. Specifically, the four-stage labels were derived by grouping N1 and N2 as “light sleep,” with N3 representing “deep sleep”.
- CPAP Models: Trained using CPAP-related signals to predict sleep stages, including two-stage (wake/sleep), three-stage (wake/REM/NREM), and four-stage (wake/REM/light/deep) classification.

### 2.2. Performance Evaluation Protocol

Pretrained models for each input modality, developed on separate private datasets, were directly applied to the Duke dataset for inference and ensemble evaluation. Performance was assessed using accuracy, Cohen’s kappa (κ), and macro F1-score, which capture overall correctness, agreement beyond chance, and balanced precision–recall under class imbalance. Additionally, class-wise metrics including sensitivity (recall), positive predictive value (PPV, precision), specificity, and F1 were reported to evaluate the reliability of detection across all sleep stages, with particular attention to underrepresented stages such as REM.

## 3. Model

### 3.1. Feature Extraction for the TipTraQ Model

The TipTraQ model was trained using data collected from the TipTraQ device, which includes both PPG and IMU signals sampled at 50 Hz. To ensure optimal PPG signal quality and mitigate temperature-induced vasoconstriction, participants were equipped with the TipTraQ device during CPAP therapy sessions conducted in a controlled clinical environment. This environment was maintained at a standard ambient temperature of approximately 25 °C with typical indoor humidity levels. Data are initially acquired by the TipTraQ device and transmitted via Bluetooth to a paired smartphone. Subsequently, the data are uploaded to a cloud-based server for asynchronous, offline processing, where the sleep-staging models are executed to generate predictions. The feature extraction and signal processing pipeline were implemented in Python 3.11, leveraging standardized libraries including NeuroKit2 (v0.2.13) [40] for physiological signal analysis, SciPy (v1.13.1) for digital filtering, and NumPy (v1.26.4) for high-performance numerical computations. Four features were derived for model training: the PPG envelope, instantaneous heart rate (IHR), blood oxygen saturation (SpO_2_), and actigraphy (ACTG).

Pre-processing of PPG. The raw PPG signals consisted of three optical channels: green, red, and infrared. First, a Butterworth filter was applied to the PPG signal to isolate the frequency band of interest and suppress noise. The filtering was implemented using a zero-phase forward–backward filter to avoid phase distortion. Next, a moving average smoothing was applied to remove residual high-frequency fluctuations, effectively performing a detrending operation that mitigates baseline wander and slow drifts in the PPG waveform. After baseline drift removal from each channel, the signals were jointly processed for artifact suppression by highlighting the alignment of three channels. Peak detection was then applied to all three channels using the maximum-slope algorithm, and the detected peaks were fused to obtain a robust peak-to-peak interval for IHR estimation. In addition, the PPG amplitude, referred to as the PPG envelope [41], was extracted as a respiration-related feature that indirectly reflects breathing patterns. The PPG envelope reflects respiratory modulations; it provides information for identifying the irregular respiratory patterns characteristic of REM sleep and the stabilized respiration observed during deep sleep.

Derivation of IHR. Given the detected maximal slope timestamps $\left\{t_{1},t_{2},\ldots ,t_{n}\right\}$ from the pre-processing step, the IHR at the *i*-th cardiac cycle was defined as

(1) $$\mathrm{IHR}\left(t_{i}\right)=\frac{60}{t_{i}-t_{i-1}},\;i=2,\ldots ,n.$$

The resulting set $S_{\mathrm{IHR}}:=\left\{(t_{i},\mathrm{IHR}\left(t_{i}\right))\right\}$ represents a nonuniform sampling of the underlying heart rate. Monotonic cubic spline interpolation was then applied to $S_{\mathrm{IHR}}$ and resampled at 4 Hz to obtain a continuous IHR signal. This feature serves as a proxy for autonomic nervous system (ANS) activity, which exhibits distinct shifts across sleep stages, most notably the reduced HRV during the deep sleep stage due to parasympathetic dominance [42,43].

Derivation of SpO_2_. SpO_2_ was computed from the red and infrared PPG channels [3], providing an additional physiological indicator relevant to sleep stage transitions. SpO_2_ provides vital information regarding sleep-disordered breathing; it is essential for detecting nocturnal desaturation events, which occur exclusively during sleep and are particularly prevalent in patients with REM-related AHI elevations [44].

Derivation of ACTG feature. The IMU data were used to derive ACTG features, which capture movement patterns correlated with sleep stages. To quantify the fingertip ACTG activity, we computed an adaptive version of the Euclidean Norm Minus One (ENMO) [45] from the three-axis acceleration signal. Instead of subtracting the fixed 1g gravitational baseline, we subtracted the median Euclidean norm value for each recording. This approach accounts for individual differences and variations in device placement, emphasizing movement-related deviations while reducing baseline bias. The resulting signal is denoted as

$$a\left(t\right)={\left[a_{x}\left(t\right),a_{y}\left(t\right),a_{z}\left(t\right)\right]}^{⊤}\in R^{3}\;,$$

where t∈R, and its $L_{2}$-norm was computed as

(2) $$a_{\mathrm{norm}}\left(t\right)={\left(|a_{x}{\left(t\right)|}^{2}+|a_{y}{\left(t\right)|}^{2}+{\left|a_{z}\left(t\right)\right|}^{2}\right)}^{\frac{1}{2}}.$$

After downsampling $a_{\mathrm{norm}}$ to 4 Hz, the ACTG feature was obtained by subtracting the median of $a_{\mathrm{norm}}\left(t\right)$ and taking the absolute value:

(3) $$\mathrm{ACTG}\left(t\right)=\left|\;a_{\mathrm{norm}}\left(t\right)-\mathrm{median}\;\left(a_{\mathrm{norm}}\left(t\right)\right)\right|.$$

ACTG remains the most direct and reliable feature for differentiating wakefulness from sleep, as it captures the gross motor inactivity necessary for sleep onset.

All the above features were temporally aligned to a sampling rate of 4 Hz for PPG model training.

### 3.2. Feature Extraction for the CPAP Model

The CPAP model was trained using data collected from CPAP machines, including CPAP-airflow and positive airway pressure (PAP) signals sampled at 25 Hz. Same as the TipTraQ model feature extraction setting, the feature extraction and signal processing pipeline were implemented in Python 3.11, leveraging standardized libraries including NeuroKit2 (v0.2.13) for physiological signal analysis, SciPy (v1.13.1) for digital filtering, and NumPy (v1.26.4) for high-performance numerical computations.

Pre-processing of CPAP-airflow. CPAP-airflow was first detrended by mean removal and then band-pass-filtered using a second-order Butterworth filter (0.1–0.35 Hz) to isolate respiratory rhythms while suppressing low-frequency drift and high-frequency noise. The CPAP-airflow serves as the core metric for monitoring breath-by-breath morphology; it enables the identification of highly stable, rhythmic breathing patterns characteristic of deep sleep, as well as the abrupt, irregular respiratory rhythms typically observed during REM sleep.

Derivation of IRR. Breath intervals were estimated by detecting local maxima corresponding to exhalation onsets. Let $\left\{t_{1},t_{2},\ldots ,t_{n}\right\}$ denote the timestamps of successive exhalation peaks. The instantaneous respiratory rate (IRR) at the *i*-th breathing cycle was then computed as

(4) $$\mathrm{IRR}\left(t_{i}\right)=\frac{60}{t_{i}-t_{i-1}},\;i=2,\ldots ,n.$$

The resulting set $S_{\mathrm{IRR}}:=\left\{(t_{i},\mathrm{IRR}\left(t_{i}\right))\right\}$ was interpolated using a monotonic cubic spline and resampled at 25 Hz to yield a continuous respiratory rate signal. IRR is a critical diagnostic marker for distinguishing REM from NREM stages, while a gradual decrease in overall respiratory rate provides a reliable indicator of sleep onset.

Integration of PAP. PAP signals represent the pressure delivered by the CPAP device over time. Although smoother and less dynamic, they encode pressure fluctuations associated with respiratory effort and sleep stage transitions, which reflect changes in upper airway muscle tone and the degree of collapse. This change is most notable during REM sleep, where muscle atonia often necessitates a compensatory increase in therapeutic pressure, which can be helpful for the model to learn REM sleep better. The PAP signal was combined with CPAP-airflow and IRR features to enrich the model’s input representation.

All CPAP features were subsequently aligned and downsampled to 1 Hz, as respiratory dynamics during sleep vary more slowly than cardiac dynamics, making higher temporal resolution unnecessary.

### 3.3. Training and Validation Overview

**Figure 1:**
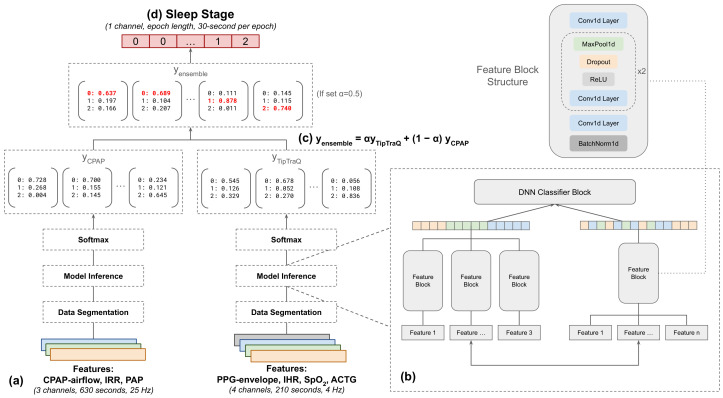
A systematic overview of the proposed method. In part (**a**), CPAP and TipTraQ features are fed into two separately trained models, with data segmentation customized for each input type. Both models share the same architecture, detailed in part (**b**). In part (**c**), the softmax probability outputs from each model are combined through an ensemble process to obtain the final stage probability for each epoch. Finally, in part (**d**), the sleep stage with the highest probability is selected as the final classification.

The overall ensemble framework, comprising the TipTraQ and CPAP models, is illustrated in Figure 1. The TipTraQ and CPAP models were trained independently to predict sleep stages over each 30 s epoch as described below. Each model is trained to predict either two sleep stages (Wake and Sleep), three sleep stages (Wake, REM, and NREM), or four sleep stages (Wake, REM, Light and Deep).

The TipTraQ model was trained using data collected at Taipei Veterans General Hospital (VGHTPE), where participants with suspected OSAHS underwent overnight PSG sleep studies while wearing a TipTraQ device. The model was trained on four input features, including PPG envelope, IHR, SpO_2_, and ACTG, segmented into 210 s windows and resampled at 4 Hz. Additional details regarding the database and model validation are provided in [31].

The CPAP model was trained using data collected at Yale New Haven Hospital and Taiwan Integrated Database for Intelligent Sleep (TIDIS) https://tidis.org/en/ (accessed on 1 March 2023), where participants with previously diagnosed OSAHS underwent in-laboratory, attended positive airway pressure titration PSG. The model incorporated CPAP-airflow, IRR, and PAP signals, segmented into 630 s windows and resampled at 1 Hz. Further details regarding the database and model validation can be found in [17].

Both models are pretrained and designed to capture temporal dependencies within their respective input streams using a one-dimensional convolutional neural network (1D-CNN) backbone [46], followed by fully connected layers for sleep stage classification. Each model generated a softmax probability distribution over two, three, or four sleep stages. These probability distributions were subsequently used in the ensemble framework that will be detailed in the next subsection. The training objective was to minimize the categorical cross-entropy loss between the predicted and true sleep stages. Model parameters were optimized using the Adam optimizer with an initial learning rate of 1 × 10^−4^ and a batch size of 512. Early stopping based on the validation F1-score was employed to mitigate overfitting. To address the severe class imbalance, particularly for the deep sleep stage, we utilized weighted loss functions during training to penalize misclassifications of minority classes. The model is implemented in PyTorch (v2.5.1) and trained on NVIDIA A100 GPUs, with each experiment repeated across multiple random seeds for reproducibility.

The validation protocol comprises two distinct evaluations. First, to assess our model’s primary performance, we conducted direct inference and ensemble on the Duke dataset, where the total test case is N=32. Second, to account for potential variability across clinical centers, device types, and patient populations, we performed a 3-fold cross-validation (CV). In this scheme, the Duke dataset was partitioned into three folds based on subject ID to prevent data leakage. The modality-specific models were fine-tuned on the training folds and subsequently evaluated on the respective testing folds to ensure a robust assessment of generalization.

### 3.4. Ensemble Method

After the two modality-specific models were trained, their softmax probability predictions were temporally synchronized to the same 30 s epoch resolution. The ensemble was conducted at the prediction level, combining the softmax probability vectors from both models. Specifically, let $y_{\mathrm{TipTraQ}}$ and $y_{\mathrm{CPAP}}$ denote the predicted class probability distributions from the TipTraQ and CPAP models, respectively. The final fused prediction was computed as a weighted combination:

(5) $$y_{\mathrm{ensemble}}=\alpha \;y_{\mathrm{TipTraQ}}+\left(1-\alpha \right)\;y_{\mathrm{CPAP}},$$

where α∈[0,1] is the ensemble coefficient controlling the contribution of each modality. The final value of α was set to 0.5 in our reported results, which means we considered the weights from the two outputs equally. The selection of the α weight was validated through empirical optimization via a sensitivity analysis conducted on the 3-fold CV results.

This late-fusion soft-voting scheme helps preserve the integrity of each signal type. In practice, different modalities possess different sampling rates and noise characteristics, among others. Late fusion allows each modality-specific model to optimize feature extraction independently, which avoids the complexities of synchronization and potential feature-interference often encountered in early or feature-level fusion. This architecture ensures modality independence, which is a key requirement for stable long-term monitoring, where sensor-specific artifacts should not propagate across modalities.

### 3.5. Post-Processing

After obtaining the ensemble output, a post-processing step was applied to the stage-wise predictions to produce smoother and physiologically plausible sleep stage sequences. First, we follow the suggestion in [26] to avoid REM sleep during the initial 60 min of the recording, as normal REM latency in healthy adults typically ranges from 90 to 120 min [47]. This 60 min REM-exclusion window serves as a physiological constraint to ensure that the predicted hypnograms are clinically plausible and free from transient artifacts during the sleep-onset period. While internal validation indicated that this constraint provides only marginal quantitative improvements in overall accuracy, it was retained to align the model’s output with standard clinical expectations and to suppress spurious REM detections prior to the first true sleep cycle.

Subsequently, each sleep stage was smoothed using a connected component-based algorithm. This approach implements a 1D morphological closing operation using a flat structuring element of size 2k+1, where *k* represents the kernel radius. In this context, two disjoint segments of the same target stage are considered “connected” if the temporal gap between them does not exceed the structural threshold defined by the kernel. By merging these proximal segments, the algorithm functions as a distance-based filter that effectively performs gap filling, ensuring temporal continuity and suppressing short-term fluctuations [48]. It is applied sequentially in the order of REM followed by waking. Since waking and REM sleep exhibit similar physiological patterns that can confuse the model, this post-processing step was applied sequentially to reduce misclassifications. Specifically, REM stages were smoothed first to prevent wake predictions from appearing within a REM segment, followed by the smoothing of wake stages. This approach preserves physiologically plausible transitions while reducing spurious predictions.

### 3.6. Signal Quality Mask

To mitigate potential motion artifacts resulting from patient discomfort or nocturnal movements, a multi-stage suppression logic was implemented. First, the PPG signals were processed via a zero-phase Butterworth band-pass filter to eliminate baseline wander and high-frequency interference without introducing phase distortion. Second, a Signal Quality Index (SQI) was employed to evaluate data integrity: for the TipTraQ model, SQI was derived from the perfusion index [49] (calculated from red and infrared light ratios), while for the CPAP model, artifacts were identified by detecting periods where the airflow amplitude exhibited near-zero oscillations, signifying signal corruption. During training, these SQI masks were utilized to guide the model, ensuring that feature extraction was prioritized within high-fidelity segments. Furthermore, our probabilistic ensemble framework inherently mitigates localized noise; when a specific modality (e.g., fingertip PPG) exhibits low confidence due to transient artifacts, the system dynamically shifts its weights toward the stable respiratory patterns provided by the CPAP machine, maintaining overall classification robustness.

## 4. Results

All results are derived from the direct inference validation protocol, except Section 4.4, where a complementary analysis based on 3-fold CV is provided to further demonstrate the model’s robustness.

### 4.1. Evaluation of Two-Stage Classification

As shown in Table 4, for the two-stage results, both TipTraQ and CPAP models got higher PPV in detecting the wake stage but exhibited lower sensitivity. After ensembling, the wake stage performance was balanced by combining outputs from both, even with a higher sensitivity. Notably, the ensemble F1-score of 0.678 for the wake stage represents a substantial improvement of 27% and 16% compared to the TipTraQ (0.534) and CPAP (0.584) baselines, respectively.

### 4.2. Evaluation of Three-Stage Classification

**Figure 2:**
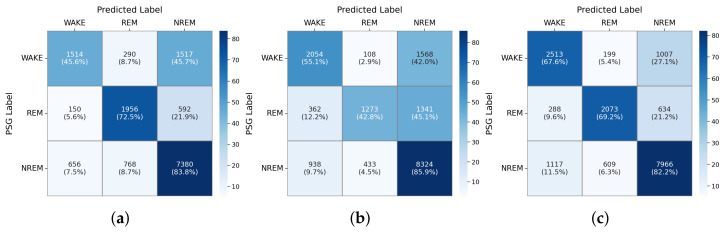
The direct inference of (**a**) TipTraQ, (**b**) CPAP, and (**c**) ensemble 3-stage classification confusion matrices on the Duke dataset.

Based on the data in Table 5 and the confusion matrices (Figure 2), the ensemble model achieves a superior Cohen’s κ of 0.587. Error analysis shows that the ensemble strategy effectively reconciles complementary markers to resolve modality-specific misclassifications. In REM detection, the integration of PPG-derived autonomic features reduced the “REM-as-NREM” error from 45.1% (CPAP model) to 21.2%, overcoming the masked breathing irregularity under CPAP pressure. Conversely, respiratory stability from the CPAP signal refined REM precision by curbing the “Wake-as-REM” confusion observed in the TipTraQ model (reduced from 8.7% to 5.4%).

The most significant gain occurred in wakefulness detection, where the “Wake-as-NREM” confusion, which is a major failure mode for both single-modality models (42.0–45.7%), was markedly resolved. By leveraging simultaneous respiratory instability and hemodynamic/motion markers, the ensemble model increased wake accuracy to 67.6%. These results confirm that the ensemble approach fundamentally integrates distinct physiological signals to resolve ambiguous state transitions rather than merely smoothing the output.

Note that performance remains lower than PPG-only models from the SOTA in Table 1 trained on non-CPAP treatment cohorts. We hypothesize that it is due to the attenuation of physiological variability under CPAP therapy. See Section 5.4 for further discussion.

### 4.3. Evaluation of Four-Stage Classification

Table 6 presents the performance metrics of four-stage results for a subset of eight subjects who exhibited deep sleep. Within this cohort, the TipTraQ model demonstrated higher sensitivity (0.421) for deep sleep detection, whereas the CPAP model achieved a superior PPV (0.370). By leveraging our ensemble strategy, we attained a sensitivity of 0.596 and a PPV of 0.373 for the deep sleep stage. Notably, the resulting F1-score of 0.459 represents an improvement of over 30%, a performance level comparable to current SOTA methods.

To address the limited sub-cohort size (N=8) for deep sleep, we provided performance metrics with 95% confidence intervals (CIs) estimated via bootstrap resampling, as detailed in Table 7. Despite the inherent individual variations in OSAHS patients, the ensemble model achieved a higher Kappa 0.411 (95% CI: 0.286–0.539) and deep sleep F1-score 0.353 (95% CI: 0.167–0.560) compared to the individual modalities. While the wide CIs for deep sleep sensitivity (0.198–0.747) reflect the expected high inter-subject variability in deep sleep patterns among CPAP users, the ensemble approach consistently refined the precision of deep sleep detection, mitigating the high false-positive rates (PPV) often observed in single-modality outputs.

**Figure 3:**
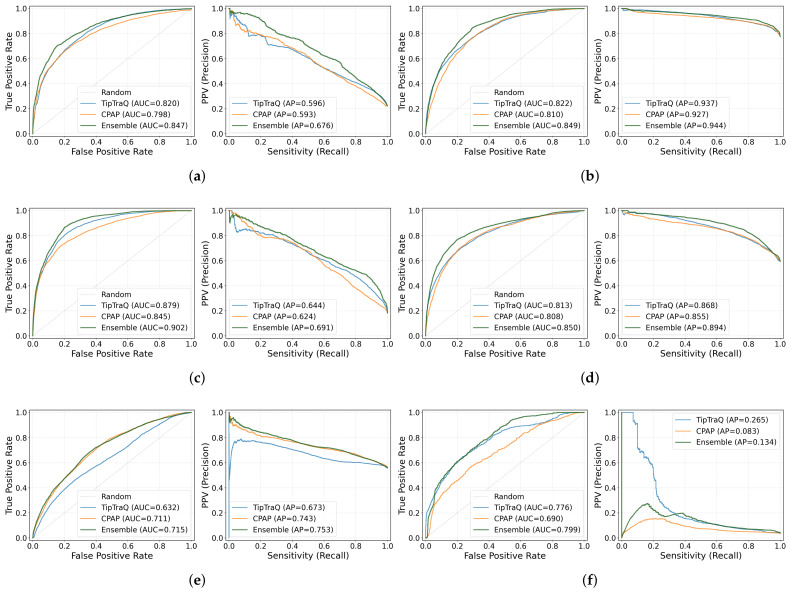
Comparative performance analysis via ROC and PRC. The figure evaluates the classification efficacy of direct inference TipTraQ (blue), CPAP (orange), and the Ensemble model (green) across three task granularities: (**a**,**b**) 2-stage (Wake versus Sleep), (**c**,**d**) 3-stage (REM versus NREM), and (**e**,**f**) 4-stage (Light versus Deep sleep) classifications. In each sub-figure, the left panel presents the ROC curve, while the right panel illustrates the PRC.

The efficacy of the imposed weighted loss in identifying infrequent deep sleep stages is quantified through the Receiver Operating Characteristic (ROC) curve and the Precision–Recall curve (PRC), which are shown in Figure 3. A consistent trend is observed where the multimodal ensemble model encapsulates the performance envelopes of the individual TipTraQ and CPAP models across most sleep stages. Recall that PRC analysis is more informative than overall accuracy for imbalanced medical datasets.

### 4.4. Model Robustness via Cross-Validation

Table 4, Table 5, Table 6 and Table 7 present a comparison between direct inference and 3-fold CV results, the latter of which evaluates model performance following fine-tuning on the Duke dataset. Our observations indicate that fine-tuning enhances the capability of both models to detect the wake stage; consequently, the ensemble model’s wake recall and overall Cohen’s Kappa outperform those achieved via direct inference. For three-stage classification, the REM sensitivity and PPV achieved a more balanced distribution across both models, indicating increased predictive reliability. However, the 3-fold CV F1-scores for the REM and deep sleep stages in both three- and four-stage classifications were lower than the direct inference results. This discrepancy may stem from data sparsity during the fine-tuning process, because the CPAP titration recordings in this split-test cohort are, on average, half the duration of standard PSG tests, and patient discomfort associated with CPAP use often results in suppressed REM and deep sleep cycles. As demonstrated in the tables, the low standard deviation (SD) across all metrics indicates that the model’s performance remains consistent across different folds. This minimal variance suggests that the predictions are well-balanced and further underscores the robustness of our proposed method.

### 4.5. Qualitative Visualization of Hypnograms

**Figure 4:**
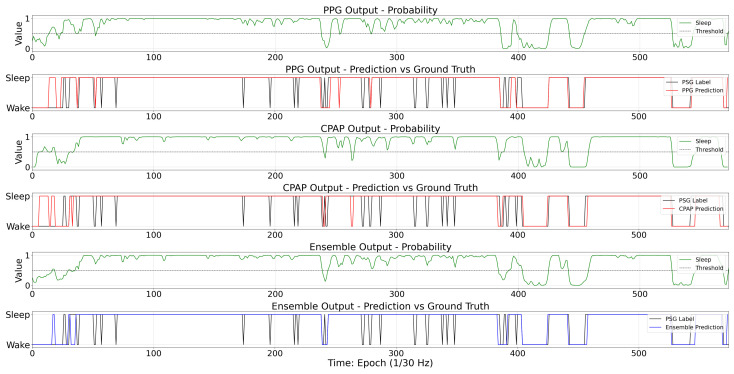
Representative two-stage classification direct inference results. Each panel shows overnight hypnograms with PSG labels, predicted labels, and stage probabilities determined by the PPG model, the CPAP model, and the ensemble outputs. This case achieved an overall accuracy of 0.911, a kappa value of 0.718, and a macro F1-score of 0.859.

**Figure 5:**
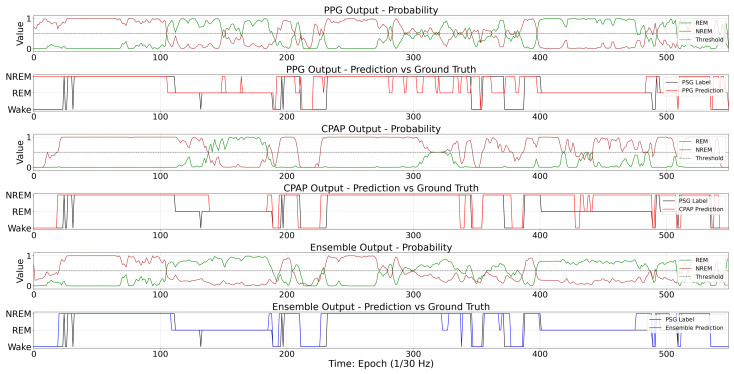
Representative three-stage classification direct inference results. Each panel shows overnight hypnograms with PSG labels, predicted labels, and stage probabilities determined by the PPG model, the CPAP model, and the ensemble outputs. This case achieved an overall accuracy of 0.922, a kappa value of 0.807, and a macro F1-score of 0.876.

**Figure 6:**
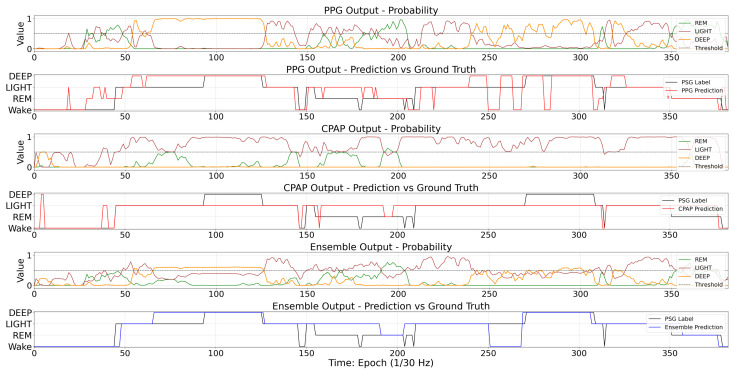
Representative four-stage classification direct inference results. Each panel shows overnight hypnograms with PSG labels, predicted labels, and stage probabilities determined by the PPG model, the CPAP model, and the ensemble outputs. This case achieved an overall accuracy of 0.858, a kappa value of 0.589, and a macro F1-score of 0.712.

Next, we present an illustration of the two-stage prediction results in Figure 4 for visualization. We found that the TipTraQ model and the CPAP model detect highly overlapping wake segments; therefore, the final ensemble result exhibits a similar trend to each individual model. For the three-stage prediction results in Figure 5, note that the TipTraQ model and CPAP model detected distinct portions of REM sleep in both cases. By ensembling the outputs, the final predictions provided a more complete prediction of REM sleep, closely aligned with the PSG-labeled ground truth. A similar observation can be found in the four-stage classification shown in Figure 6, where both signal sources enhanced the final ensemble results for the detection of deep sleep fragments more completely. This result suggests the usefulness of fusing information from different channels.

### 4.6. Sensitivity Analysis of the Ensemble Weight

**Figure 7:**
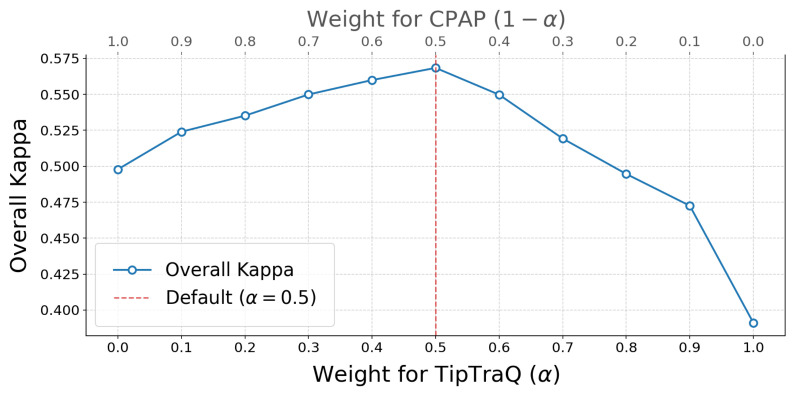
Sensitivity analysis of the ensemble weight α on the overall Cohen’s Kappa. The bottom *X*-axis represents the weight assigned to the TipTraQ model (α), while the top X-axis indicates the corresponding weight for the CPAP model (1−α). The 3-stage classification results, derived from 3-fold cross-validation, show that the highest predictive performance is achieved at the default value of α=0.5 (dashed red line), demonstrating that an equal-weight integration of both modalities is optimal for sleep stage classification.

Table 8 and Figure 7 present the sensitivity analysis of the ensemble weight α. To determine the optimal weight, α was varied from 0 to 1 in increments of 0.1, with performance evaluated using the mean metrics from the 3-fold CV. The empirical results indicate that the highest overall performance is achieved at α=0.5. This suggests that a balanced integration of both models provides the most robust and accurate insights for final sleep stage prediction.

### 4.7. Prediction Confidence Analysis

Since the stage classification output is determined by the class with the largest ensemble softmax probability, the corresponding probability values can be grouped into different confidence levels (or probability ranges). For each confidence level, we analyze the classification accuracy of the corresponding epochs as well as the mean predicted confidence of the model.

**Figure 8:**
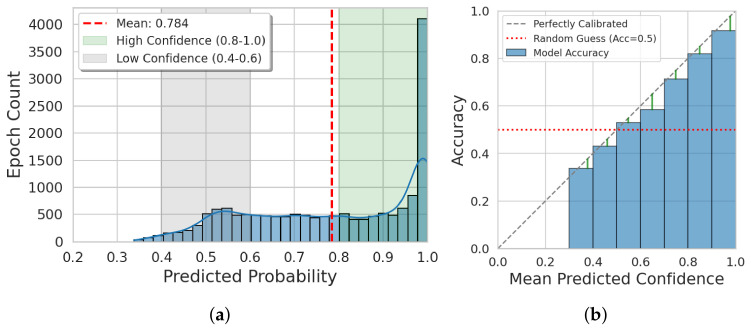
Confidence and reliability analysis of using the 3-stage ensemble model on the Duke dataset (direct inference). (**a**) Distribution of predicted probabilities, where the high mean confidence (0.784) indicates that the ensemble model consistently produces decisive predictions. (**b**) Reliability diagram comparing mean predicted confidence against observed accuracy. The proximity of the model accuracy (blue bars) to the perfectly calibrated line (dashed diagonal) demonstrates high predictive reliability and well-calibrated probability outputs across different confidence intervals.

Figure 8a shows the distribution of predicted probabilities, with an average predicted probability of 0.784, indicating that the model is neither overconfident nor underconfident. Figure 8b presents the reliability diagram [50] of the proposed method, which shows that predictions with a mean confidence below 0.6 generally achieve an accuracy lower than 0.5, which is close to random guessing.

Furthermore, Table 9 groups the predicted probabilities into three confidence levels: High (0.8–1.0), Intermediate (0.6–0.8), and Low (0.3–0.6). The table summarizes the performance of the ensemble model under different levels of prediction confidence, reflecting how well the model performs when it is more or less “certain” about its predictions. A clear positive correlation between confidence level and classification performance is observed. In the high-confidence regime, the model demonstrates strong reliability, achieving an accuracy of 0.893. In contrast, performance degrades substantially in the low-confidence regime, where accuracy drops to 0.501, which, in the context of a three-stage classification task, is only marginally better than random guessing.

Table 10 presents the calibration analysis across different probability ranges. The Expected Calibration Error (ECE) is defined as a weighted average of the absolute difference between accuracy and confidence across all bins [51]:

(6) $$ECE=\sum_{m=1}^{M}\frac{|B_{m}|}{n}\left|\mathrm{acc}\left(B_{m}\right)-\mathrm{conf}\left(B_{m}\right)\right|,$$

where *M* denotes the number of bins (with M=7 in Table 10), $|B_{m}|$ is the number of samples in the *m*-th bin (corresponding to the “Epoch Count”), *n* is the total number of samples (i.e., the sum of all epoch counts), $\mathrm{acc}(B_{m})$ represents the classification accuracy within bin *m*, and $\mathrm{conf}(B_{m})$ denotes the mean predicted confidence of samples in bin *m*. The resulting ECE of 0.045 is generally considered low, indicating good calibration and a strong alignment between the model’s predicted probabilities and its actual performance.

## 5. Discussion

### 5.1. Synergistic Potential of Multimodal Fusion

The proposed ensemble model demonstrates improved performance in three-stage sleep classification compared with standalone CPAP-based [17] and TipTraQ-based [31] models. By leveraging the complementary strengths of these modalities, the ensemble approach particularly enhances detection of the REM stage. Physiologically, the CPAP model captures respiratory dynamics and airway stability [18], while the TipTraQ model reflects autonomic cardiovascular modulation [5]. Together with IMU signals for motion detection [4], these modalities provide a comprehensive representation of sleep macro-structure, enabling more accurate classification even in the presence of single-modality signal degradation.

### 5.2. Classification Accuracy and Error Distributions

**Figure 9:**
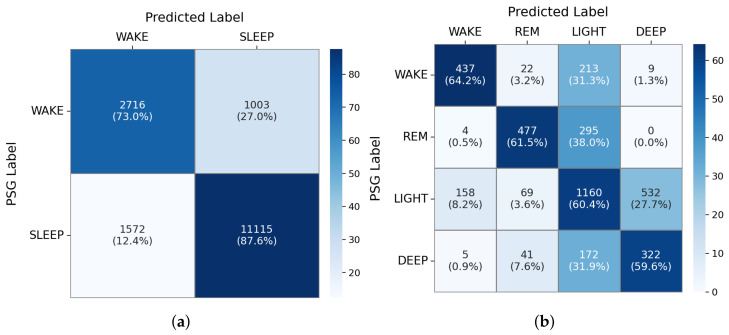
The (**a**) 2-stage and (**b**) 4-stage classification confusion matrices of ensemble direct inference results from the Duke dataset. Note that the 4-stage analysis (**b**) was conducted on a subset of 8 subjects (*N* = 3916 epochs), whereas (**a**) and Figure 2c include the full dataset (*N* = 16,406 epochs).

Figure 2 shows the confusion matrices for the three-stage classification results from the different models, which comprise the main results of this work. Figure 9 shows additional confusion matrices for the two- and four-stage classifications. The results achieved high diagnostic accuracy in the two-stage (wake/sleep) task, with true positive rates of 73.0% and 87.6%, respectively. As classification complexity increased to three-stage and four-stage models, the ensemble maintained robust performance, particularly for the NREM and Light sleep categories, while effectively balancing sensitivities across REM and Wake stages.

In the two-stage (Wake versus Sleep) task (Figure 3a,b), the ensemble model achieved an AUC of 0.847 and 0.849, respectively, indicating a robust baseline for basic sleep–wake differentiation. Notably, the PRCs reveal that the ensemble strategy significantly maintains higher precision even at high-recall levels compared to single-sensor approaches.

The benefit of modality fusion is most pronounced in the three-stage (REM versus NREM) classification (Figure 3c,d). For the REM stage, the ensemble model reached an AUC of 0.902 and an Average Precision (AP) of 0.691, representing a substantial improvement over the standalone CPAP (AP = 0.624) and TipTraQ (AP = 0.644) models. This suggests that the integration of cardiovascular dynamics and respiratory patterns effectively resolves the ambiguity between REM and other states.

For the four-stage classification (Figure 3e,f), while detecting minority classes like N3 remains challenging, the ensemble model maintains a superior ROC profile (AUC = 0.799). The PRC analysis for deep sleep highlights a trade-off. Although TipTraQ exhibits high precision at very low recall, the ensemble model provides a more balanced trajectory, mitigating the high false-positive rates observed in the CPAP-only model.

Collectively, these results confirm that the ensemble approach does not merely aggregate data but achieves a synergistic effect that enhances classification reliability across all levels of sleep-stage granularity.

### 5.3. Challenges in Deep Sleep Detection and Data Sparsity

A primary challenge in this study is the granular classification of deep sleep. In our cohort of 32 subjects, only 8 exhibited observable N3 stages. This scarcity is likely attributed to the “first-night effect” or the fragmented sleep architecture common in severe OSAHS patients undergoing CPAP titration [52]. While the low PPV (0.373) for deep sleep suggests limitations in current slow-wave sleep detection, our 3-fold CV results indicate a robust trend of improvement through fusion compared to single-modality inference. The wide confidence intervals reported underscore the necessity for future large-scale studies to better characterize deep sleep morphology using non-EEG markers. Future iterations will investigate data augmentation for minority classes and the inclusion of cardiac entropy features to enhance stage-specific discrimination.

### 5.4. Impact of the CPAP Titration Environment

Compared with existing results listed in Table 1, particularly those based solely on PPG sensors, the performance of the proposed model appears comparatively lower, despite incorporating additional modalities such as CPAP-airflow and IMU signals. However, it is important to note that most state-of-the-art PPG-based approaches are developed and evaluated using standard PSG recordings obtained without CPAP intervention. In contrast, our data were collected during CPAP titration, a clinical setting in which normal sleep dynamics are inherently perturbed [52]. CPAP alters respiratory mechanics, stabilizes upper-airway airflow, and reduces sleep-disordered breathing events, thereby attenuating respiratory variability that is otherwise informative for sleep stage discrimination. It has been found that heart rate variability is reduced in sleep apnea patients receiving CPAP treatment [53]. Moreover, the process of pressure adjustment, mask discomfort, and frequent arousals during titration can fragment sleep architecture and blur physiological distinctions between sleep stages. Jointly, the multimodal signals in CPAP-treated sleep may exhibit reduced stage-specific contrast compared with PSG recordings acquired under natural sleep conditions, which partially explains the observed performance gap relative to PPG-only models trained on non-CPAP datasets.

### 5.5. Model Robustness and Domain Adaptation

Regarding data consistency, minor discrepancies in total epoch counts across models arise from sensor-specific signal disconnections. A key clinical advantage of our framework is its resilience; the system remains functional when one modality is compromised. Therefore, normalized confusion matrices provide a more reliable performance comparison than raw epoch counts. To prevent underfitting with the N=32 cohort, our late-fusion architecture enables independent feature extraction for each modality before integration, thereby preserving minority class characteristics.

As shown in Section 4.4, the implementation of 3-fold cross-validation and fine-tuning effectively mitigates the impact of “domain shift” arising from different hardware and patient populations. By recalibrating pretrained weights to the Duke dataset, the model adapts to center-specific characteristics. Furthermore, our sensitivity analysis of the ensemble weight α (Figure 7) demonstrates that an equal weighting (α=0.5) consistently yields optimal performance. This “inverted U-curve” empirically validates that the TipTraQ and CPAP signals contribute complementary information that is most effectively captured when integrated with balanced priority. While a static weighting of α=0.5 provides a robust and transparent baseline, it may not account for real-time signal degradation. Future work will explore adaptive reliability control where α is dynamically adjusted based on the SQI, allowing the model to favor the modality with higher instantaneous signal quality.

### 5.6. Clinical Utility: Titration Efficacy and REM Detection

Accurate sleep staging during CPAP therapy provides potential clinical benefits. While EEG remains the gold standard, its use during CPAP treatment is limited by physical interference between EEG leads and CPAP masks, often resulting in reduced patient compliance. This limitation underscores the need for less intrusive sensing approaches, such as integrated PPG or CPAP-derived signals. Continuous measurement of CPAP-related changes in sleep architecture, such as decreased slow-wave and REM sleep and increased arousals, may enable the evaluation of treatment efficacy beyond conventional respiratory indices and support individualized therapy [52]. Improved detection of REM sleep is particularly relevant for CPAP titration. REM sleep is characterized by reduced upper-airway muscle tone and increased respiratory instability, making it critical for determining the pressure required to maintain airway patency. More accurate identification of REM periods may help prevent under-titration in patients with REM-dominant OSAHS [54,55], improve characterization of REM-associated hypoxemia, and inform optimal pressure settings. For AutoPAP users, enhanced sleep stage estimation through a multi-sensor approach using the TipTraQ, or other similar devices, may also enable smoother, more responsive pressure adjustments, which will be investigated in future work.

While we do not explore sleep quality in this work, the current work has potential applications in continuously monitoring sleep quality for patients receiving CPAP therapy. It is known that monitoring sleep quality trajectories over time, which correlate with reductions in daytime sleepiness and improvements in cognitive function [11,12], may enhance adherence and long-term outcomes in OSAHS populations [56]. Sleep stage metrics and continuity measures derived from diagnostic and titration polysomnography have been linked to subsequent CPAP usage, indicating that sleep staging can serve as a valuable marker for monitoring and predicting treatment adherence [57]. Moreover, because CPAP can improve broader health markers such as quality of life and oxygen saturation, evaluating objective sleep stage changes provides a more complete picture of therapeutic benefits than respiratory measures alone [58].

### 5.7. Adaptive Reliability and Quality Masking

Regarding future usability and clinical integration, our SQI-masking logic provides a foundational framework for adaptive reliability control. While the current study demonstrates that the ensemble model effectively leverages cross-modal redundancy, we will further design the dynamic weighting mechanism in future iterations.

Specifically, the SQI derived from both the TipTraQ perfusion index and CPAP-airflow stability can serve as a “gatekeeper” for diagnostic output. In scenarios where both sensors exhibit poor signal quality simultaneously (e.g., during intense physical movement or sensor displacement), the system could be programmed to withhold predictions rather than provide low-confidence results. This “fail-safe” strategy ensures that the clinical report remains high-fidelity and prevents the accumulation of erroneous data during periods of total signal corruption.

By integrating such an artifact-aware veto mechanism, the proposed framework not only enhances the robustness of multi-modal sleep staging but also moves toward a more user-centric design. This allows for a balance between sophisticated data collection and the practical constraints of long-term patient compliance in home-based CPAP therapy.

### 5.8. Probabilistic Estimates as a Confidence Metric

We shall also discuss the sleep-stage probabilities produced by each model, as shown in Figure 5 and Figure 6. Inaccurate classifications tend to occur when the predicted probability is close to 50%, indicating increased model uncertainty. These probability estimates may assist clinicians in manually reviewing and correcting automatically determined sleep stages. For example, in the confidence analysis in Section 4.7, we observe that predictions with a mean confidence below 0.6 generally achieve an accuracy lower than 0.5, which is close to random guessing. For such cases, we suggest that these probability estimates can serve as a reliable confidence metric. For cases with low confidence (e.g., below 0.6), the system can automatically flag segments that require further clinical attention, thereby streamlining the review process rather than increasing the manual workload. When extra information is available, experts can perform manual editing to refine the final output.

Our preliminary experiments demonstrate that even without joint training, combining independently trained models through ensembling can substantially improve sleep stage classification performance. This result highlights the synergistic potential of the simple ensemble multimodal fusion. To avoid overfitting, in this work, we focus on validating the feasibility of fusing two existing single-modality models. From a technical perspective, we can further decompose PPG, IMU, and CPAP-airflow signals to better design features. This possibility will be explored in our future work.

### 5.9. Limitations and Future Directions

Several limitations warrant consideration. First, the requirement for an additional PPG device may limit user compliance; future efforts should aim for comparable accuracy using CPAP-derived signals alone. Second, while our N=32 cohort provides preliminary validation, larger multi-center studies are essential to ensure generalizability across different CPAP devices and home environments. Last but not least, the black-box nature of CNNs is well recognized, and, while accumulating empirical evidence is necessary, it remains insufficient without a clear theoretical foundation, which we consider essential for ensuring scientific rigor and patient safety. Future work will focus on cross-center validation and model interpretability, as well as exploring new features to improve the detection of slow-wave sleep.

## 6. Conclusions

This study presents an ensemble framework that integrates PPG- and CPAP-based models for automatic sleep stage classification. While each modality captures distinct physiological dynamics—cardiovascular and respiratory, respectively—their combination provides complementary information that enhances stage discrimination. Experimental results obtained from the Duke dataset demonstrate that the ensemble approach significantly improves overall performance compared to single-modality models, particularly in the detection of REM and wake stages. These findings suggest that leveraging multimodal physiological signals can yield more robust and accurate sleep monitoring, even when models are trained separately. Future work will focus on collecting synchronized PPG–CPAP datasets and developing unified multimodal architectures for end-to-end training.

## Figures and Tables

**Table 3:** Overall demographic and clinical characteristics of the Duke dataset.

Characteristic	Mean ± SD	Median (Q1–Q3)
Age	52.8 ± 14.8	52.0 (41.0–65.25)
BMI	39.4 ± 11.2	36.99 (30.28–44.58)
AHI 1A Rule	57.0 ± 38.1	42.85 (32.65–62.15)
AHI 1B Rule	42.1 ± 38.2	25.48 (19.83–49.96)
TST	197.9 ± 54.6	196.0 (161.50–234.75)
Sex	N	%
Male	18	56
Female	14	44
Race	N	%
White	23	72
African American	9	28
Cardiovascular Comorbidities	N	%
Hypertension	5	16
Arrhythmia	1	3
Medication Use	N	%
Beta Blocker	8	25
Hypnotics	4	13
Antidepressants	12	38

**Table 4:** Overall results of two-stage classification on the Duke dataset compared with TipTraQ and CPAP baseline models. Acc: Accuracy; κ: Cohen’s kappa; F1: macro F1-score. Results are reported as the mean ± SD across the 3-fold cross-validation.

	Overall			Sensitivity		PPV		Specificity		F1	
Model	Acc	κ	F1	Wake	Sleep	Wake	Sleep	Wake	Sleep	Wake	Sleep
Direct Inference											
TipTraQ	0.823	0.429	0.713	0.454	0.930	0.650	0.855	0.930	0.454	0.534	0.891
CPAP	0.821	0.470	0.735	0.555	0.899	0.616	0.873	0.899	0.555	0.584	0.886
Ensemble	0.843	0.576	0.787	0.730	0.876	0.633	0.917	0.876	0.730	0.678	0.896
3-Fold Cross-Validation											
TipTraQ	0.757 ± 0.03	0.432 ± 0.02	0.710 ± 0.02	0.745 ± 0.07	0.758 ± 0.06	0.501 ± 0.02	0.906 ± 0.01	0.758 ± 0.06	0.745 ± 0.07	0.597 ± 0.01	0.823 ± 0.04
CPAP	0.833 ± 0.01	0.538 ± 0.02	0.769 ± 0.01	0.646 ± 0.04	0.890 ± 0.02	0.651 ± 0.01	0.890 ± 0.01	0.890 ± 0.02	0.646 ± 0.04	0.648 ± 0.02	0.890 ± 0.01
Ensemble	0.854 ± 0.01	0.613 ± 0.01	0.806 ± 0.00	0.753 ± 0.06	0.885 ± 0.03	0.678 ± 0.03	0.921 ± 0.01	0.885 ± 0.03	0.753 ± 0.06	0.711 ± 0.01	0.902 ± 0.01

**Table 5:** Overall results of three-stage classification on the Duke dataset compared with TipTraQ and CPAP baselines. Acc: Accuracy; κ: Cohen’s kappa; F1: macro F1-score. Results are reported as the mean ± SD across the 3-fold cross-validation.

	Overall			Sensitivity			PPV			Specificity			F1		
Model	Acc	κ	F1	Wake	REM	NREM	Wake	REM	NREM	Wake	REM	NREM	Wake	REM	NREM
Direct Inference															
TipTraQ	0.821	0.511	0.676	0.456	0.725	0.838	0.653	0.649	0.778	0.930	0.913	0.650	0.537	0.685	0.807
CPAP	0.807	0.452	0.636	0.551	0.428	0.859	0.612	0.702	0.741	0.897	0.960	0.566	0.580	0.532	0.795
Ensemble	0.843	0.587	0.730	0.676	0.692	0.822	0.641	0.720	0.829	0.889	0.940	0.756	0.658	0.706	0.826
3-Fold Cross-Validation															
TipTraQ	0.761 ± 0.02	0.416 ± 0.03	0.610 ± 0.02	0.745 ± 0.07	0.526 ± 0.02	0.631 ± 0.08	0.501 ± 0.02	0.541 ± 0.00	0.794 ± 0.01	0.758 ± 0.06	0.900 ± 0.01	0.776 ± 0.04	0.597 ± 0.01	0.533 ± 0.01	0.701 ± 0.05
CPAP	0.800 ± 0.01	0.486 ± 0.01	0.660 ± 0.01	0.646 ± 0.04	0.600 ± 0.00	0.753 ± 0.05	0.651 ± 0.01	0.541 ± 0.05	0.781 ± 0.01	0.890 ± 0.02	0.883 ± 0.02	0.713 ± 0.03	0.648 ± 0.02	0.568 ± 0.03	0.766 ± 0.02
Ensemble	0.827 ± 0.01	0.552 ± 0.01	0.704 ± 0.01	0.753 ± 0.06	0.585 ± 0.02	0.783 ± 0.06	0.678 ± 0.03	0.642 ± 0.03	0.803 ± 0.01	0.885 ± 0.03	0.925 ± 0.01	0.736 ± 0.04	0.711 ± 0.01	0.611 ± 0.01	0.791 ± 0.03

**Table 6:** Overall results of 4-stage classification on the Duke dataset compared with TipTraQ and CPAP baselines. Acc: Accuracy; κ: Cohen’s kappa; F1: macro F1-score; W: Wake; R: REM; L: Light; D: Deep. Statistics are derived from the sub-cohort of subjects exhibiting deep sleep (N=8). Results are reported as the mean ± SD across the 3-fold cross-validation.

	Overall			Sensitivity				PPV				Specificity				F1			
Model	Acc	κ	F1	W	R	L	D	W	R	L	D	W	R	L	D	W	R	L	D
Direct Inference																			
TipTraQ	0.782	0.369	0.539	0.520	0.669	0.580	0.421	0.550	0.667	0.674	0.270	0.912	0.923	0.719	0.814	0.535	0.668	0.624	0.329
CPAP	0.776	0.287	0.464	0.597	0.335	0.731	0.170	0.479	0.676	0.575	0.370	0.864	0.960	0.482	0.954	0.532	0.448	0.644	0.233
Ensemble	0.806	0.432	0.611	0.642	0.615	0.604	0.596	0.724	0.783	0.630	0.373	0.948	0.958	0.659	0.840	0.680	0.689	0.617	0.459
3-Fold Cross-Validation																			
TipTraQ	0.801 ± 0.00	0.378 ± 0.02	0.542 ± 0.01	0.727 ± 0.05	0.322 ± 0.03	0.742 ± 0.07	0.334 ± 0.10	0.732 ± 0.02	0.640 ± 0.04	0.592 ± 0.01	0.399 ± 0.01	0.939 ± 0.01	0.954 ± 0.01	0.531 ± 0.06	0.921 ± 0.02	0.729 ± 0.03	0.427 ± 0.02	0.657 ± 0.04	0.356 ± 0.05
CPAP	0.802 ± 0.00	0.401 ± 0.02	0.497 ± 0.01	0.905 ± 0.03	0.479 ± 0.04	0.696 ± 0.03	0.045 ± 0.02	0.604 ± 0.04	0.599 ± 0.03	0.640 ± 0.01	0.160 ± 0.08	0.864 ± 0.01	0.918 ± 0.02	0.639 ± 0.06	0.963 ± 0.01	0.724 ± 0.03	0.530 ± 0.01	0.666 ± 0.01	0.069 ± 0.04
Ensemble	0.829 ± 0.00	0.465 ± 0.03	0.558 ± 0.02	0.877 ± 0.05	0.451 ± 0.07	0.809 ± 0.05	0.118 ± 0.03	0.744 ± 0.01	0.665 ± 0.02	0.658 ± 0.02	0.312 ± 0.04	0.929 ± 0.02	0.942 ± 0.01	0.609 ± 0.08	0.958 ± 0.01	0.804 ± 0.02	0.534 ± 0.04	0.724 ± 0.01	0.169 ± 0.03

**Table 7:** Performance metrics for deep sleep detection, reported as Mean (95% CI). Statistics are derived from the sub-cohort of subjects exhibiting deep sleep (N=8).

	Overall			Deep Sleep (N3 Stage) Specific			
Model	Acc	κ	F1	Sensitivity	PPV	Specificity	F1
Direct Inference							
TipTraQ	0.748 (0.693–0.793)	0.281 (0.159–0.414)	0.472 (0.340–0.569)	0.490 (0.229–0.746)	0.274 (0.141–0.402)	0.823 (0.754–0.892)	0.346 (0.174–0.508)
CPAP	0.801 (0.773–0.828)	0.343 (0.235–0.454)	0.470 (0.378–0.562)	0.171 (0.026–0.344)	0.283 (0.103–0.492)	0.967 (0.932–0.995)	0.175 (0.045–0.327)
Ensemble	0.810 (0.768–0.842)	0.411 (0.286–0.539)	0.561 (0.471–0.657)	0.475 (0.198–0.747)	0.287 (0.136–0.433)	0.849 (0.788–0.912)	0.353 (0.167–0.560)
3-Fold Cross-Validation							
TipTraQ	0.793 (0.731–0.831)	0.343 (0.220–0.456)	0.458 (0.338–0.559)	0.249 (0.054–0.498)	0.259 (0.060–0.493)	0.955 (0.911–0.992)	0.235 (0.038–0.456)
CPAP	0.813 (0.789–0.836)	0.391 (0.268–0.498)	0.488 (0.394–0.570)	0.087 (0.017–0.188)	0.276 (0.059–0.550)	0.968 (0.925–1.000)	0.119 (0.027–0.243)
Ensemble	0.836 (0.815–0.857)	0.443 (0.326–0.554)	0.532 (0.452–0.617)	0.143 (0.013–0.333)	0.223 (0.017–0.453)	0.972 (0.933–0.995)	0.169 (0.019–0.366)

**Table 8:** Sensitivity analysis of the ensemble weight α based on 3-fold cross-validation. Performance is evaluated using overall accuracy, Cohen’s kappa, and macro F1-score of the 3-stage classification.

α	0.0	0.1	0.2	0.3	0.4	0.5	0.6	0.7	0.8	0.9	1.0
Acc	0.812	0.824	0.829	0.835	0.838	0.840	0.831	0.818	0.806	0.796	0.754
κ	0.498	0.524	0.535	0.550	0.560	0.568	0.550	0.519	0.495	0.473	0.391
F1	0.668	0.685	0.693	0.703	0.710	0.712	0.697	0.676	0.659	0.645	0.587

**Table 9:** Comparison of 3-stage classification performance, stratified by model-predicted confidence levels. The data illustrate the positive correlation between the model’s self-reported confidence and its empirical predictive quality.

Confidence Level	Acc	F1	Confidence	Epoch Count
High (0.8–1.0)	0.893	0.837	0.946	8416
Intermediate (0.6–0.8)	0.649	0.647	0.699	4251
Low (0.3–0.6)	0.501	0.492	0.523	3502

**Table 10:** Calibration analysis across probability ranges, comparing observed accuracy with mean predicted confidence. The Expected Calibration Error (ECE) is reported to quantify the overall alignment.

Probability Range	Acc	Confidence	Epoch Count
0.3–0.4	0.337	0.377	199
0.4–0.5	0.430	0.460	1004
0.5–0.6	0.530	0.548	2498
0.6–0.7	0.585	0.650	2142
0.7–0.8	0.714	0.749	2109
0.8–0.9	0.819	0.850	2076
0.9–1.0	0.917	0.977	6340
Final ECE: 0.045			

## Data Availability

The dataset analyzed in this study originates from a private clinical trial and contains sensitive personal health information. Due to ethical and legal restrictions imposed by the Institutional Review Board of Duke University Hospital, the data cannot be shared publicly or upon request.
